# Matrix Metalloproteases as Influencers of the Cells’ Social Media

**DOI:** 10.3390/ijms20163847

**Published:** 2019-08-07

**Authors:** Daniel Young, Nabangshu Das, Anthonia Anowai, Antoine Dufour

**Affiliations:** 1 Department of Physiology and Pharmacology, University of Calgary, Calgary, AB T2N 4N1, Canada; 2 McCaig Institute for Bone and Joint Health, University of Calgary, Calgary, AB T2N 4N1, Canada; 3 Faculty of Kinesiology, University of Calgary, Calgary, AB T2N 4N1, Canada; 4 Department of Biochemistry and Molecular Biology, University of Calgary, Calgary, AB T2N 4N1, Canada

**Keywords:** matrix metalloproteinases (MMPs), protease, inflammation, signaling, invasion, apoptosis, chemokine, cytokine, proteomics, interferon

## Abstract

Matrix metalloproteinases (MMPs) have been studied in the context of cancer due to their ability to increase cell invasion, and were initially thought to facilitate metastasis solely through the degradation of the extracellular matrix (ECM). MMPs have also been investigated in the context of their ECM remodeling activity in several acute and chronic inflammatory diseases. However, after several MMP inhibitors failed in phase III clinical trials, a global reassessment of their biological functions was undertaken, which has revealed multiple unanticipated functions including the processing of chemokines, cytokines, and cell surface receptors. Despite what their name suggests, the matrix aspect of MMPs could contribute to a lesser part of their physiological functions in inflammatory diseases, as originally anticipated. Here, we present examples of MMP substrates implicated in cell signaling, independent of their ECM functions, and discuss the impact for the use of MMP inhibitors.

## 1. Introduction: MMPs Act as Emojis in Cell–Cell Communication

Matrix metalloproteinases (MMPs) have been associated with multiple inflammatory diseases [1,2,3], but the initial hypothesis that their proteolytic functions were solely linked to extracellular matrix (ECM) remodeling is outdated and needs to be extended to include additional substrates such as chemokines, cytokines, and cell surface receptors [4,5,6,7,8,9]. The catalytic activity of MMPs is tightly regulated by the tissue inhibitors of metalloproteinases (TIMPs), and is a key contributor in the overall outcome of substrate processing [10]. Interestingly, MMPs can also induce cell–cell communications through a non-proteolytic and non-ECM manner. For example, MMP2 was demonstrated to induce cell migration and signaling under static mechanical strain [11]. Increased mechanosensing properties in osteocytes was observed through decreased MT1–MMP expression [12]. Thus, MMPs are multi-tasking proteins that play key roles in cellular interactions and signaling; similar to emojis in our messages and social media posts, MMPs are able to tune and modulate cellular communications. Unfortunately, both emojis and MMPs are often misunderstood. Here, we describe examples of MMP substrates that are implicated in cell-to-cell communication and their physiological connection to inflammatory diseases.

## 2. MMPs Process the Gatekeepers of Cell Signaling

**Figure 1:**
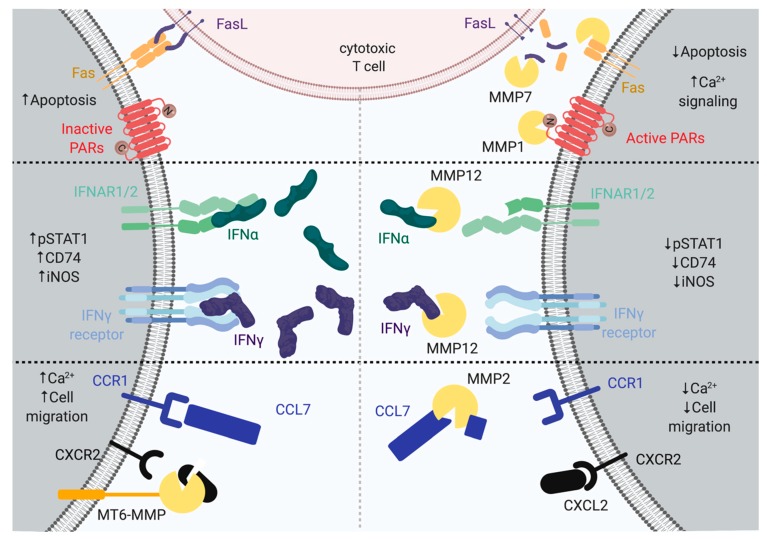
*Upper*, matrix metalloproteinase 1 (MMP1) cleavage of protease-activated receptors (PARs) result in increased Ca^2+^ signaling. MMP7 processing of tumor necrosis factor ligand superfamily member 6 (FASL) leads to decreased apoptosis. The right side displays MMP7 proteolysis on tumor necrosis factor receptor superfamily member 6 (FAS), the CD95 ligand (FasL), and PARs, leading to decreased apoptosis and increased Ca^2+^ activation. *Middle*, MMP12 can cleave interferon-alpha (IFNα) and interferon-gamma (IFNγ) at the C-terminus, leading to the termination of the tyrosine-protein kinase JAK1-signal transducer and activator of transcription 1-alpha/beta (JAK–STAT1) signaling pathway. The right side displays MMP12 proteolysis on IFNα and IFNγ, leading to the decreased phosphorylation of STAT1, HLA class II histocompatibility antigen gamma chain (CD74), and inducible nitric oxide synthase (iNOS). *Lower*, MMP2 cleaves C-C motif chemokine 7 (CCL7), and MT6-MMP cleaves C-X-C motif chemokine 2 (CXCL2), resulting in decreased migration and Ca^2+^ activation. The left side displays MT6-MMP proteolysis on CXCL2, leading to increased cell migration and Ca^2+^ activation. The right side displays MMP2 proteolysis on CCL7, leading to decreased cell migration and Ca^2+^ activation. MMPs are depicted as yellow Pacman.

Cell surface receptors act as gatekeepers of cell signaling networks, as they provide the integral component that allows extracellular messages to be converted to intracellular signals. Proteolytic regulation is a key physiological process, as the cleavage of surface receptors can impact their activation state and half-life, and alter normal downstream transduction of receptor signals. Protease-activated receptors (PARs) are a family of G-protein coupled receptors (GPCRs) that are activated via a proteolytic mechanism [13]. They exist on most cell types, including macrophages, smooth muscle cells, and endothelial cells [14], and play roles in platelet aggregation, adhesion, cytokine production, and migration through the activation of different G proteins (G_s,_ G_q_, and G_12/13_) [14,15]. PAR1 is cleaved into its active form by thrombin, but also by other proteases including tryptase, neutrophil elastase, and MMP1 through its N-terminal extracellular exodomain (Figure 1) [13]. This occurs at a unique peptide sequence between ^41^R↓S^42^ (Table 1), liberating an N-terminal fragment that functionally interacts with the C-terminal region of the exodomain on PAR1 and triggers signal transduction from the GPCR (Figure 1). In cancer cells, thrombin cleavage of PAR1 increases tumor cell adhesion to matrix components, including platelets and vascular epithelial cells [16]. In platelets, PAR1 forms a heterodimer with PAR4, which is an isoform with lower affinity for thrombin. Thrombin cleavage of the surface receptor activates G_q_-driven and G_13_-driven pathways, leading to increased intracellular calcium and platelet aggregation [16]. MMP1 and MMP13 were found to be highly expressed in platelets, and are converted to their catalytically active form in the presence of fibrillar collagen [15]. MMP1 can also cleave membrane-tethered PAR1 at the *N*-terminal exodomain to generate signal transduction, albeit in a non-classical manner compared to thrombin–PAR1 cleavage [17]. Functional analysis involving the replacement of single amino acids around the cut site of PAR1 determined that MMP1’s cleavage site is unique and specific to MMP1’s active site [15]. Using a synthetic peptide matched to the exodomain sequence, the cleavage site was identified between ^39^D↓P^40^, which corresponds to a fragment of two amino acids that is distal to the thrombin cleavage site. MMP1 processing of PAR1 induces signaling, resulting in changes in morphology, activation via an alternate pathway, and phosphorylation of the p38 protein [13]. Overall, MMP1 can modulate PAR signaling in a cell-dependent manner, leading to various outcomes in downstream cell signaling.

## 3. MMP7 Modulates CD95/Fas Signaling in Cell Death

CD95, apoptosis-mediating surface antigen tumor necrosis factor receptor superfamily member 6, (FAS)-mediated apoptosis is an extrinsic contact-dependent cell death pathway that is used by the adaptive immune system to eradicate neoplastic or infected cells [48]. The CD95 ligand (tumor necrosis factor ligand superfamily member 6/FASL) is expressed on the surface of cytotoxic T lymphocytes, which seek out target cells expressing the CD95 receptor [49]. Upon the association of a T cell with its cognate Fas receptor, apoptosis is initiated via recruitment of the adapter protein, Fas-associated death domain (FADD), procaspases, and a caspase regulator to form the death-inducing signaling complex (DISC), resulting in cell death [30,48,49]. Inhibitors that increase FasL expression at cell surfaces are able to terminate tumor cell proliferation [50]. Subverting Fas-mediated cell death signals allows cancer cells to develop resistance to chemotherapy drugs and increases chances of tumor survival. Some methods of Fas-related apoptosis evasion include occupation of the Fas receptor by a soluble FasL antagonistic ligand, the downregulation of Fas receptor expression, the cleavage of FasL ligand by MMP7 [51], and cleavage of the Fas receptor by MMP7 [29] (Figure 1). The extent to which MMP7 contributes to cell death escape via cleavage of the Fas/CD95 receptor was investigated in a previous study using the recombinant CD95 protein [29]. MMP7 was shown to cleave CD95 when on the surface of HT-29 colon cancer cells and prevented apoptosis, while treatment with a broad spectrum MMP inhibitor increased cell sensitivity to CD95-mediated death [29]. MMP7 has been shown to cleave FasL, producing a fragment from the soluble FasL ligand that is able to initiate apoptosis via Fas [52]. In contrast, others have suggested that the MMP7 cleavage of FasL inhibits the tumor killing action of cytotoxic T cells [51]. Importantly, Fas/CD95 processing is cell type-dependent, and further examination is needed to clarify the mechanism. Thus, MMP7 activity could be responsible for multiple mechanisms of immune defense evasion, in a cell-dependent manner, via the Fas/FasL system.

## 4. Co-Expression of MMP14/MT1–MMP and DR6 in Cell Death

Death receptor 6 (DR6) is a member of the tumor necrosis factor receptor (TNFR) family, which is ubiquitously expressed in humans, with higher occurrence in the heart, brain, and lymphoid organs [53,54]. Among its structural features are four highly conserved extracellular cysteine-rich domains that form the ligand binding site. This site is an intracellular death domain that is homologous to that of other members of the death receptor subfamily, as well as numerous post-translational modifications, including glycosylation, which can influence DR6 trafficking and interactions [53,55]. DR6 expression can be increased via activation of the NF-κB pathway in tumor cells; however, the overexpression of ectopic DR6 can activate the NF-κB and JNK pathways, and lead to apoptosis [54]. A cognate ligand is yet to be identified for DR6, and has generated speculations leading to discoveries regarding the role of DR6 in healthy tissues and pathologic conditions. Interestingly, the amyloid precursor protein (APP) was recently suggested as a major agonist for DR6 based on the ability of cleaved N-terminal APP fragments to bind DR6 [56]. DR6 is implicated in the degeneration of neurons in Alzheimer’s disease via caspase-mediated apoptosis [56], the development and differentiation of hematopoietic cells [54,57], and immune system modulation [54,57]. In addition, DR6 is widely expressed on the surface of tumor cells, particularly in prostate and breast cancer [57]. This suggests unconventional interactions of pro-apoptotic DR6 in neuronal inflammation in comparison to cancer cells that may alter apoptosis, but no clear mechanism has been described yet. Explorative proteomics experiments have identified DR6 as a substrate of MMP14/membrane type 1 matrix metalloproteinase (MT1–MMP) [58]. The ectodomain of DR6 spans amino acids 67 to 211, and MT1–MMP cleavage releases the entire extracellular portion to create a soluble DR6 fragment [53,58]. MT1–MMP cleavage of DR6 ectodomain diverted T cell differentiation away from Th1, induced monocyte cell death, and affected the cytokine profiles of immature dendritic cells [57]. The extent to which DR6 cleavage influences physiological conditions in other tissues remains unclear; however, this suggests interesting functions of DR6 and MT1–MMP in boosting the innate and adaptive immune response in anti-tumor therapy.

## 5. Processing of Ephrin B2 Receptor Mediates Cell Motility

Receptor tyrosine kinase ephrin type-B receptor 2 (EPHB2) and its associated ligand ephrin-B2 (EFNB2) are both transmembrane proteins whose interactions guide embryonic neuronal development [59], specifically the spatial organization of cellular networks in the nervous system [60]. They are part of the larger Eph–ephrin family, whose roles also include directing cell boundary division, the morphogenesis of vasculature, and axon guidance [61]. There are two groups within this family: ephrin A-type ligands that connect to the cell membrane via a glycosylphosphatidylinositol anchor (GPI) to which Ephrin A receptors bind, and ephrin B-type ligands that are transmembrane proteins that associate with Eph B receptors [59,60,61]. The responses of receptor–ligand are enhanced when a large cluster of proteins interact at the contact site [61]. Activation at the contact site occurs in the ligand-bearing and receptor-bearing cells, which are termed reverse and forward signaling respectively, and is thus bi-directional [62]. Forward signaling (Eph-expressing cell) activates tyrosine kinase activity and the downstream phosphorylation of members of the Rho family GTPases that modulate intracellular morphological changes [61]. Mutations or the loss of ephrin B ligands impacts the reverse signaling capabilities, and in mice, it may alter the migration of capillary, lymphatic, and neuronal networks [61]. Ephrin-B2 activates the EphB2 receptor on the surface of neighboring cells to trigger cell adhesion and repulsion, thereby directing cell movements. The processes that allow repulsion and attraction between cells involves the remodeling of the cytoskeleton as well as actin polymerization [63]; however, the mechanism by which high-affinity binding of the surface-bound receptor and ligand is translated into repulsion remains uncharacterized. One proposed mechanism is that the specific cleavage of the EphB2 extracellular domain by MMP7 and MMP9 alter ephrin-B2-induced activation [28]. Two distinct large and short fragments of the EphB2 ectodomain were isolated from murine hippocampal neurons and 14-day-old mice embryonic brains. This cleavage was inhibited by an MMP2/MMP9 inhibitor, suggesting the involvement of MMPs in ephrin-B2-directed shedding of the EphB2 ectodomain [28]. MMP7 and MMP9 can cleave endogenous and recombinant EphB2 in vitro and on the surface of HEK-293 cells and hippocampal neurons to generate two fragments of lengths matching those originally discovered [28]. Further investigation revealed two sites at which MMPs targeted the EphB2 receptor: between ^394^N↓I^395^ and ^432^D↓L^433^ (Table 1). Amino acid substitution at these sites rendered mutant Eph2 insusceptible to MMP cleavage and thus failed to illicit cell–cell repulsion, although ephrin-B2 could still bind to the receptor [28]. One of these sites was conserved amongst all the Eph receptors, while the other was only expressed in EphB2 and EphB3, suggesting that repulsive signaling from Eph receptors depends on the predisposition of the receptor to MMP cleavage [28].

## 6. Processing of CD44 Decreases Cell Adhesion

Tumor invasion and metastasis are key processes of cancer progression that require cells to enhance their migratory ability [64,65,66]. CD44, a ubiquitous transmembrane receptor, is necessary for cell adhesion, wound healing, tumor invasion, and cell migration due to its broad-spectrum ligand interactions. CD44 interacts with ECM components, cytokines, and growth factors [22,67,68]. Its ability to facilitate multiple binding interactions with ECM components may promote the establishment of the cellular niche [69]. CD44 is the primary cognate receptor for hyaluronate, which is a molecule that aids in migration by inducing changes in the ECM to promote cell movement [70]. CD44 is derived from a single gene, but has multiple variants due to alternative splicing in 10 of its 19 exons [67]. Common in the structure of CD44 are post-translational N-glycosylations and O- glycosylations, and phosphorylation within the ectodomain, which might determine CD44 receptor potency [71]. Multiple CD44 variants are markers of stem cells and cancer stem cells, and have been linked to angiogenesis and increased metastatic activity in tumors [22,67,72]. However, findings of increased soluble CD44 (sCD44) in patients with aggressive cancers suggest that additional post-translational modifications, such as proteolytic processing, may play essential roles in CD44’s mechanism of action [22,72]. Additionally, CD44 maintains adhesive interactions to other ECM components that have to be detached in order for migration to occur [67]. MT1–MMP is a key protease that is implicated in increasing cell migration and invasion and interacts with CD44 at the cell surface in the direction of migration [72]. Kajita et al. analyzed and confirmed the processing of CD44 by MT1–MMP in cancer cell lines [22]. The co-expression of MMP-14 and CD44 in human breast carcinoma cells revealed measurable amounts of 70-kDa fragments and a significant decrease in hyaluronate binding ability [22,70]. Three ~26-kDa sized CD44 fragments were generated at ^162^R↓T^163^, ^186^R↓S^187^, and ^192^G↓Y^193^ by MT1–MMP [22]. The blade I of the MT1–MMP hemopexin domain was shown to interact with CD44, resulting in the phosphorylation of the epidermal growth factor receptor (EGFR) [72]. This interaction was blocked with peptides mimicking blade I of the MT1–MMP hemopexin domain, leading to decreased cancer cell metastasis in a mouse model of breast cancer and a reduction in new blood vessel formation in a chorioallantoic membrane assay in chickens [72]. Thus, CD44–MT1–MMP interactions may be responsible for migration, invasion, metastasis, and angiogenesis through proteolytic modulation and impact on downstream signaling.

## 7. MMP Processing of Chemokines

Chemokines are essential regulators in the immune system that act on G protein-coupled receptors (GPCRs) [73,74,75]. There are four main types, which are classified by the structure of a conserved cysteine-containing motif at the amine terminus, including the C, CC, CXC, and the CX3C motifs (C represents a cysteine and X represents any amino acid) [76]. Chemokine receptors are involved in multiple biological processes, including angiogenesis, wound healing, virus sensing, cell signaling, calcium activation, and hematopoiesis (Figure 1) [76,77]. Immune cells respond to multiple chemokines, and are thus subjected to this complex landscape of immune responses and signaling networks. For example, the binding of CXCL12 to CXCR4 results in the development and maturation of several immune cell linages including B cells, monocytes, natural killer cells, neutrophils, and macrophages [77]. Antagonists to CXCR4 generate abnormal numbers of neutrophils in peripheral circulation [78]. CCL7 attracts monocytes and eosinophils, but not neutrophils, via interactions with CCR1, CCR2, or CCR3 [79]. CCL7 acts as a ligand for CCR2, which is required for monocyte mobilization out of the bone marrow [80]. CCL13 attracts lymphocytes, monocytes, basophils, and eosinophils, but not neutrophils via CCR2B or CCR3, resulting in downstream signaling and chemotaxis [79,81]. To date, little is known about the post-translational modification of chemokines. Several proteases, including MMPs, are capable of cleaving chemokines at their *N*-terminus or *C*-terminus to alter their function, resulting in the formation of haptotactic gradients that help direct cell migration [6,9]. The establishment of potent chemical gradients occurs through interactions between peptide sequences of various chemokines and unique glycoproteins known as glycosaminoglycans (GAGs) [76]. GAGs are present in the ECM and on the surface of endothelial cells where they can facilitate the efficient infiltration of inflammatory cells into tissues from the blood stream [82]. MMPs modulate the functions of CCL7 and CCL13 through precise processing (Table 2). The truncated form of both CCL7 (5–76) and CCL13 (8–75) lose agonist activity in comparison to their full-length counterparts, as demonstrated using cell migration assays [83]. Proteolytic truncations of CCL7 and CCL13 results in a dampening of the inflammatory response, as demonstrated in a mouse model of inflammatory edema [83].

Chemokines also regulate T-cell development by inducing several developmental stages that occur in the thymus. Progenitor T cells (thymocytes) are double negatives, meaning that they lack markers denoting any specialized role (CD4^-^, CD8^-^). Upon entering the thymus, they may be pushed toward a CD4^+^/CD8^-^ or CD4^-^/CD8^+^ phenotype [77]. Progression through these stages is well coordinated and occurs in various microenvironments within the thymus [91]. Initial migration to the thymus is CCR7/CCR9-dependent, and activated by CCL21 or CCL19 [92]. Once in the thymus, thymocytes move toward the cortex in response to CCL19 and CXCL12, which is mediated by CCR9 [93]. There, they receive further cues for T-cell maturation, including signals that cause the apoptosis of autoreactive T cells. CD4^+^/CD8^-^ or CD4^-^/CD8^+^ T cells also begin to express CCR7 via CCL19 signaling [94]. MMPs were demonstrated to process multiple chemokines, including CCL5, CCL19, CCL21, and CXCL12 (Table 2); however, the proteolytic effect of MMPs on these chemokines during T-cell development and adaptation has yet to be characterized, and remains unexplored. 

Membrane-type matrix metalloproteinase 6 (MT6–MMP or MMP25) is predominantly expressed by neutrophils, and has been shown to cleave 14 chemokines that are implicated in the recruitment of macrophages and monocytes during inflammatory processes (Table 2). Of these 14 chemokine substrates, CXCL2 and CXCL5 are involved in the recruitment of macrophages and monocytes [95,96]. The cleavage of CXCL2 at ^4^A↓T^5^ and CXCL5 at ^7^R↓A^8^ increases their chemotactic activity [84,97]. Ten MMPs can cleave CCL15 at six different sites (Table 2), resulting either in a decrease or increase of T cells and monocytes recruitment via interactions with chemokine receptor 1 (CCR1) [98]. MMP12 was identified as the most kinetically efficient MMP responsible for CCL15 proteolysis [6]. In addition to CCL15, several MMPs can cleave CCL23, which is a chemokine that is involved in the recruitment of monocytes, T lymphocytes, and neutrophils (Table 2). Cleaved CCL15 and CCL23 have increased binding to GAGs and induce chemotaxis [6]. The cleavage of CCL7 (MCP-3) by MMP2 dampens the immune response by preventing the additional recruitment of macrophages and lymphocytes [99]. CCL7 binds to CCR-1, CCR-2, and CCR-3, which are cell surface receptors on leukocytes, and when activated, these receptors cause an influx of calcium and leukocyte migration. MMP2 cleaves the first four amino acids in CCL7 (Table 1); cleaved CCL7 (CCL 5–76) is unable to induce calcium via either CCR-1 or CCR-2, and acts as an antagonist to the CCR-3 receptor [99]. Mice treated with cleaved CCL7 (5–76) suffered a reduction in mononuclear inflammatory cell infiltration as compared to full-length CCL7 [99]. Together, these results demonstrate that MMPs are key regulators of immune cell recruitment during various inflammatory processes. 

## 8. MMPs–Orchestrator of the Fine Tuning of Cytokine Signaling 

Cytokines are key regulators and messengers modulating the social landscape of cell-to-cell communication. Cytokine-mediated communication among lymphocytes, inflammatory cells, and hematopoietic cells initiate and organize an effective immune response [100]. Cytokine–receptor engagement triggers an intracellular signaling cascade; interestingly, most if not all cytokines can be post-translationally modified, altering their ability to bind to their respective cell surface receptors [7,8,86]. Traditionally, MMPs have been defined as ECM degrading proteases, but recent evidence has demonstrated that the majority of their substrates are not ECM-related proteins; rather, they are related to chemokine/cytokine networks, cell migration, kinase signaling, or transcription factors [4,5,101]. The altered expression of proteases is typically present in autoimmune and inflammatory diseases wherein an imbalance in homeostatic pro-inflammatory and anti-inflammatory cytokine expression and signaling is observed. For example, MMP12 was shown to affect both the expression and secretion of interferon-alpha (IFNα), which is a key cytokine implicated in regulating autoimmune diseases and viral infection [8,102]. When cells were infected with either coxsackievirus type B3 (CVB3) or respiratory syncytial virus (RSV), MMP12 was demonstrated to control INFα secretion indirectly by regulating the transcription of IκBα in the nucleus [8]. Extracellularly, MMP12 prevented aberrant IFNα-mediated signaling through proteolytic processing of the C-terminus, which is known to bind IFNα/β receptor 1, and terminated the phosphorylation of STAT1 (Figure 1) [8]. By the injecting a membrane-impermeable MMP12 inhibitor in CVB3-infected mice, extracellular MMP12 activity was inhibited. This resulted in an elevation of systemic IFN-α levels and a reduction of viral replication in the pancreas, without loss of the beneficial roles of intracellular MMP12 [8]. During a CVB3 murine infection, MMP9 was found to prevent virus propagation in the heart, promote immune infiltration and remodeling, and preserve cardiac output–product of the heart rate and the stroke volume [103]. *Mmp9*^-/-^ mice infected with CVB3 showed more severe myocardial injury, elevated the foci of infection and immune infiltrates along with increased levels of IFNβ1 and IFNγ in comparison to wild types [103]. Similar to IFNα, MMP12 was demonstrated to truncate the C-terminus of IFNγ, causing a loss of receptor binding ability and a decrease in JAK–STAT1 signaling [7]. In a murine model of acute peritonitis, IFNγ levels were elevated, leading to an increase in IFNγ pro-inflammatory protein signature (S100A8, S100A9, inducible nitric oxide synthase (iNOS), and STAT1) in *Mmp12^⁻/⁻^* as compared to the wild-type counterparts [7]. In two autoimmune murine arthritis models using two different genetic backgrounds (MRL/*lpr* and B10.RIII), increased disease severity along with elevated IFNγ-dependent pro-inflammatory protein signatures were observed in *Mmp12^⁻/⁻^* as compared to wild type [7]. 

Tumor necrosis factor alpha (TNFα) is a pro-inflammatory cytokine released by activated macrophages, CD4^+^ lymphocytes, natural killer cells, neutrophils, mast cells, and eosinophils. It is involved in the perpetuation of systemic inflammation in diseases such as in rheumatoid arthritis, Crohn’s disease, multiple sclerosis, and cancer [104]. TNFα is produced as a trimeric membrane-anchored precursor and secreted through a disintegrin and metalloproteinase domain-containing protein 17 (ADAM17)-regulated proteolytic pathway, resulting in the release of an active, soluble TNFα mediator [101,104,105]. The processing of TNFα by MMPs was demonstrated to modulate inflammatory signaling responses [87]. In a model of macrophage-mediated herniated disc resorption, macrophage MMP7 was shown to cleave TNFα and hindered macrophage infiltration into the disc [106]. Multiple MMPs (-1, -2, -3, -7, -9, -12, -14, and -17) have been shown to cleave active TNFα on the cell surface, further supporting their roles as influencers of cell signaling [58,87,107,108,109]. 

Interleukin-1 beta (IL-1β) is a cytokine that is cleaved into its active form by cellular proteases. Mature IL-1β is secreted from activated cells following caspase-1-dependent processing [110,111]. In addition to cleavage by cytosolic caspase-1, IL-1β can also be cleaved by MMP2, -3, and -9 in order to achieve a biologically mature form [112]. In a feed-forward mechanism, IL-1β induces MMP synthesis, which in turn dampens IL-1β activity. For example, the addition of MMP-1 reduced the IL-1β-dependent synthesis of prostaglandin E2 in human fibroblasts [88]. MMPs can regulate IL-1β activity by processing the soluble type II IL-1 decoy receptor (sIL-1R II) [113,114].

MMPs promote inflammation, but are also implicated in the anti-inflammatory regulation of immune responses. Transforming growth factor beta (TGF)-β, an anti-inflammatory cytokine, is processed by MMPs [90,115,116]. Initially, TGF-β is produced as a latent precursor containing two disulfide-linked short carboxy-terminal and large amino-terminal homodimers, which act as a latency-associated protein. Non-covalent association of these homodimers in the latent complex prevents receptor-ligand binding and the induction of TGF-β-mediated responses [115]. Following secretion, the latent TGF-β complex is cross-linked to the cellular membrane, forming a reservoir of latent TGF-β in the extracellular environment [115]. Several mechanisms including MMP proteolysis have been implicated in the release of mature TGF-β from the latent complex. MMP2 [90], MMP3 [116], MMP9 [90], and MT1–MMP [117] have demonstrated cleavage of the latent TGF-β complex, untethering TGF-β from the cell surface [118]. Furthermore, MMPs can release TGF-β by degrading decorin, which is a small collagen-associated proteoglycan that is known to act as a depot for TGF-β in the ECM [119]. In a feed-forward mechanism, TGF-β and IL-1β stimulate the production of MMP9 in rabbit corneal epithelial cells [120]. This induction of MMP9 by IL-1β occurs via NF-kB and AP-1-dependent pathways [121]. The characterization of signaling cascades regulated by MMP processing has been demonstrated in multiple systems and tissues, but more validation is needed to better understand these effects in vivo and their links to disease pathology. This could help identify new means to develop therapeutic modalities that restore normal cytokine interactions in inflammatory diseases. 

## 9. How Can We Stop MMPs *Influencing* the Cells’ Social Media Communications?

After the failure of broad-spectrum MMP inhibitors for the treatment of cancer and arthritic diseases, a global reassessment of their physiological functions was undertaken. We now know that (1) more than 10 MMPs can be beneficial in inflammatory diseases; (2) tissue and cellular localization are key in dictating MMP functions and substrate processing; (3) MMPs cleave more substrates than only ECM proteins; and (4) MMPs have non-proteolytic functions [3,5,10,64,65,122,123,124]. The question is now: would a selective MMP inhibitor have better success? Sela-Passwell et al. [125] demonstrated that by immunizing mice with a synthetic molecule that mimics the conserved structure of MMP2 and MMP9 catalytic zinc-histidine complex, they were able to protect mice from dextran sulfate sodium (DSS)-induced colitis both prophylactically and therapeutically. Several small molecules that selectively target the hemopexin domain of MMP9 were designed and were demonstrated to inhibit lung metastasis of MDA-MD-435 cells in a xenograft mouse model of breast cancer [65]. A selective MT1–MMP monoclonal antibody was demonstrated to be effective when used in combination with oseltamivir phosphate (Tamiflu) in an influenza virus model composed of PR8 influenza and *Streptococcus pneumonia* intra-nasal infection at sublethal doses [126]. Combination therapy demonstrated a synergistic therapeutic effect of the two inhibitors by both reducing the viral load and keeping ECM remodeling in homeostasis. A similar approach using a human Fab display phage library led to the development of DX-2400, which is a selective MT1–MMP monoclonal antibody that only inhibited the active form of the protease [127]. Efficacy was demonstrated in xenograft murine models of breast cancer using MDA-MB-231 cells and a syngeneic model using murine 4T1 cells by reducing tumor growth, metastasis, and angiogenesis; however, so far, little efficacy was demonstrated in human clinical trials [127,128]. It appears that broad-spectrum inhibition may not be ideal for long-term usage, but could be an alternative for short-term life-threatening conditions such as viral infections [8] or sepsis [129,130,131,132]. A better understanding of the specific roles of each MMP in a specific tissue and their communicating roles in diseases will pave the way for either selective MMP inhibition or targeting MMP substrates. Despite the long history of MMP inhibitors, we still have limited knowledge about these inflammatory proteases, and how they influence of the cells’ social media. 

## Figures and Tables

**Table 1:** Cell surface proteins and receptors cleaved by various MMPs. The cleavage site is indicated for each MMP.

Protein Name	MMP1	MMP2	MMP3	MMP7	MMP9	MMP11	MMP12	MMP13	MMP14/MT1–MMP	MMP16/MT3–MMP	MMP25/MT6–MMP	References
Amyloid protein precursor (APP)					^687^K↓L^689^ ^691^F↓A^692^ ^694^D↓V^695^ ^701^A↓I^702^				^579^N↓M^580^ ^687^K↓L^689^	^463^A↓M^464^ ^622^H↓S^623^ ^579^N↓M^580^ ^685^H↓Q^686^		[18,19,20,21]
CD44 antigen (CD44)									^162^R↓T^163^ ^186^R↓S^187^ ^192^G↓Y^193^			[22]
C-type lectin domain family 3 member A (CLEC3A)				^57^A↓L^58^ ^63^A↓L^64^ ^151^F↓L^152^								[23]
CX3CL1 (fractalkine)		^71^A↓L^72^ ^4^G↓M^5^										[24]
β-dystroglycan					^715^H↓L^716^							[25]
EMMPRIN/CD147									^209^P↓M^210^ ^214^N↓I^215^			[26,27]
Ephrin B2 receptor		^394^N↓I^395^ ^432^D↓L^433^			^394^N↓I^395^ ^432^D↓L^433^							[28]
Fas Receptor (FAS)				^19^E↓L^20^ ^32^N↓L^33^								[29,30]
Fibroblast growth factor receptor 1 (FGFR1)		^368^V↓M^369^										[31]
Integrin αV (CD51)									^891^D↓L^892^			[32]
Integrin β2 (CD18)					^705^A↓I^706^							[33]
Integrin-associated protein (IAP/CD47)												[34]
Intercellular adhesion molecule (ICAM)-1								^60^I↓E^61^ ^97^D↓G^98^				[35,36,37]
Laminin receptor						^115^A↓F^116^ ^133^P↓I^134^						[38]
Glutamate receptor ionotropic, NMDA 1 (NMDA receptor)				^516^E↓K^517^								[39]
Myelin-associated glycoprotein		^233^S↓M^234^ ^508^R↓L^509^		^233^S↓M^234^ ^508^R↓L^509^	^233^S↓M^234^ ^508^R↓L^509^							[40]
Protease-activated receptor-1 (PAR-1)	^41^R↓S^42^											[17]
Protein-tyrosine phosphatase receptor type Z (Ptprz)					^1625^R↓I^1626^ ^1627^G↓L^1628^							[41]
Tumor necrosis factor ligand superfamily member 11(RANKL)				^145^M↓M^146^								[42]
Semaphorin 4D												[43]
Tissue transglutaminase		^375^P↓V^376^ ^458^R↓A^459^ ^461^H↓L^462^							^375^P↓V^376^ ^458^R↓A^459^ ^461^H↓L^462^	^375^P↓V^376^ ^458^R↓A^459^ ^461^H↓L^462^		[44,45]
TRANCE/OPGL (TNF-related activation-induced cytokine/osteoprotegrin ligand)									^138^R↓F^139^ ^145^M↓M^146^			[46]
Urokinase plasminogen activator surface receptor (uPAR/CD87)			^108^T↓Y^109^				^108^T↓Y^109^				^108^T↓Y^109^ ^109^Y↓S^110^ ^111^R↓S^112^	[47]

**Table 2:** Chemokine and cytokine cleaved by various MMPs. The cleavage site is indicated for each MMP. ND = not determined.

Chemokine Name	MMP1	MMP2	MMP3	MMP7	MMP8	MMP9	MMP12	MMP13	MMP 14/MT1–MMP	MMP 25/MT6–MMP	References
CCL2	^4^S↓A^5^ ^27^A↓I^28^		^4^S↓A^5^ ^27^A↓I^28^	^4^S↓A^5^ ^67^K↓T^68^	^4^S↓A^5^	^4^S↓A^5^	^4^S↓A^5^	^4^S↓A^5^		^4^S↓A^5^	[6,9,83,84]
CCL3	^47^I↓A^48^			^8^L↓V^9^ ^47^I↓A^48^ ^63^I↓F^64^	^15^M↓A^16^	^15^M↓A^16^ ^64^F↓L^65^		^47^I↓A^48^			[6]
CCL4	^15^A↓A^16^ ^61^P↓A^62^	^5^V↓T^6^ ^6^T↓V^7^ ^44^P↓R^45^	^44^P↓R^45^	^5^V↓T^6^ ^61^P↓A^62^	^6^T↓V^7^	^9^L↓V^14^ ^61^P↓A^62^	^6^T↓V^7^		^5^V↓T^6^ ^6^T↓V^7^ ^44^P↓R^45^	^6^T↓V^7^	[6,84]
CCL5				^65^V↓T^66^				^4^S↓A^5^			[6]
CCL7	^4^S↓A^5^ ^27^G↓I^28^	^4^S↓A^5^ ^27^G↓I^28^	^4^S↓A^5^ ^27^G↓I^28^	^4^S↓A^5^ ^6^A↓L^7^		^4^S↓A^5^ ^27^G↓I^28^	^4^S↓A^5^	^4^S↓A^5^ ^27^G↓I^28^	^6^A↓L^7^ ^8^L↓C^9^ ^27^G↓I^28^	^4^S↓A^5^	[6,9,83,84,85]
CCL8	^4^S↓A^5^ ^27^G↓I^28^	^4^S↓A^5^ ^27^G↓I^28^	^4^S↓A^5^ ^27^G↓I^28^	^4^S↓A^5^ ^6^A↓L^7^		^4^S↓A^5^ ^27^G↓I^28^	^4^S↓A^5^	^27^G↓I^28^	^6^A↓L^7^ ^8^L↓C^9^ ^27^G↓I^28^	^4^S↓A^5^	[6,9,83,84]
CCL11		^9^W↓L^10^		^9^W↓L^10^				^9^W↓L^10^			[6]
CCL13	^4^S↓A^5^ ^26^D↓A^27^ A^27^↓L^28^ ^30^V↓P^31^ ^72^E↓I^73^	^3^V↓S^4^ ^4^S↓A^5^ ^9^C↓L^10^ ^74^C↓A^75^	^3^V↓S^4^ ^4^S↓A^5^ ^26^D↓A^27^ A^27^↓L^28^	^4^S↓A^5^ ^9^C↓L^10^ ^72^E↓I^73^ ^74^C↓A^75^	^4^S↓A^5^ ^72^E↓I^73^	^4^S↓A^5^ ^72^E↓I^73^	^4^S↓A^5^ ^72^E↓I^73^	^4^S↓A^5^ ^72^E↓I^73^	^3^V↓S^4^	^4^S↓A^5^ ^72^E↓I^73^	[6,9,84]
CCL14				^66^F↓I^67^ ^70^R↓G^71^			^3^I↓S^4^ ^6^A↓L^7^ ^71^G↓H^72^ ^72^H↓S^73^				[6]
CCL15	^24^I↓N^25^	^13^L↓V^14^ ^24^I↓N^25^ ^26^D↓A^27^ ^27^A↓E^28^	^16^V↓L^17^ ^24^I↓N^25^ ^27^A↓E^28^	^16^V↓L^17^ ^24^I↓N^25^ ^88^K↓G^89^	^24^I↓N^25^ ^27^A↓E^28^ ^42^V↓V^43^	^27^A↓E^28^	^24^I↓N^25^ ^27^A↓E^28^	^13^L↓V^14^ ^24^I↓N^25^ ^26^D↓A^27^ ^27^A↓E^28^	^13^L↓V^14^ ^26^D↓A^27^	^24^I↓N^25^ ^26^D↓A^27^	[6,84]
CCL16	^7^A↓L^8^ ^85^Q↓E^86^	^4^S↓E^5^ ^7^A↓L^8^ ^77^T↓N^78^	^7^A↓L^8^ ^85^Q↓E^86^	^7^A↓L^885^Q↓E^86^	^4^S↓E^5^ ^7^A↓L^8^ ^85^Q↓E^86^	^7^A↓L^8^ ^77^T↓N^78^ ^85^Q↓E^86^	^7^A↓L^8^ ^77^T↓N^78^ ^85^Q↓E^86^	^7^A↓L^8^ ^77^T↓N^78^	^4^S↓E^5^ ^7^A↓L^8^ ^85^Q↓E^86^	^4^S↓E^5^	[6,84]
CCL17				^3^P↓L^4^ ^69^G↓R^70^	^8^A↓L^9^		^3^P↓L^4^ ^69^G↓R^70^	^3^P↓L^4^			[6]
CCL23	^10^C↓L^11^ ^16^A↓L^17^ ^22^R↓V^23^ ^25^K↓D^26^ ^27^A↓E^28^ ^29^T↓E^30^	^10^C↓L^11^ ^16^A↓L^17^ ^25^K↓D^26^ ^27^A↓E^28^	^20^Q↓A^21^ ^25^K↓D^26^ ^90^G↓R^91^	^10^C↓L^11^ ^16^A↓L^17^ ^22^R↓V^23^ ^25^K↓D^26^ ^90^G↓R^91^	^10^C↓L^11^ ^16^A↓L^17^ ^25^K↓D^26^	^10^C↓L^11^ ^27^A↓E^28^	^10^C↓L^11^ ^16^A↓L^17^ ^22^R↓V^23^ ^25^K↓D^26^	^90^G↓R^91^	^13^L↓V^14^ ^29^T↓E^30^	^10^C↓L^11^ ^20^Q↓A^21^ ^25^K↓D^26^	[6,84]
IFNα							^160^L↓Q^161^ ^157^N↓L^158^				[8]
IFNβ						^25^N↓L^26^ ^29^F↓L^30^ ^30^L↓Q^31^ ^107^N↓L^108^ ^114^N↓L^115^					[86]
IFNγ							^136^E↓L^115^ ^157^M↓L^158^				[7]
Tumor necrosis factor (TNF)	^ND^	^ND^	^ND^	^ND^		^ND^	^ND^		^69^L↓I^70^ ^72^P↓L^73^		[58,87]
IL-1β		^141^E↓L^142^									[88]
TGFβ		^ND^				^ND^			^ND^		[89,90]
